# Hailey-Hailey disease: clinical, diagnostic and therapeutic update^☆^

**DOI:** 10.1016/j.abd.2023.12.003

**Published:** 2024-05-23

**Authors:** Adriana Maria Porro, Camila Arai Seque, Denise Miyamoto, Diego Vanderlei Medeiros da Nóbrega, Milvia Maria Simões e Silva Enokihara, Claudia Giuli Santi

**Affiliations:** a Department of Dermatology, Escola Paulista de Medicina, Universidade Federal de São Paulo, São Paulo, SP, Brazil; b Department of Dermatology, Faculty of Medicine, Hospital das Clínicas, Universidade de São Paulo, São Paulo, SP, Brazil; c Department of Pathology, Escola Paulista de Medicina, Universidade Federal de São Paulo, São Paulo, SP, Brazil

**Keywords:** Benign familial, Genetics, Genetic diseases, Inborn, Pemphigus

## Abstract

Hailey-Hailey disease is a rare genodermatosis described in 1939, with an autosomal dominant inheritance pattern, characterized by compromised adhesion between epidermal keratinocytes. It has an estimated prevalence of 1/50,000, with no gender or race predilection. It results from a heterozygous mutation in the *ATP2C1* gene, which encodes the transmembrane protein hSPA1C, present in all tissues, with preferential expression in keratinocytes. Mutations in the *ATP2C1* gene cause changes in the synthesis of junctional proteins, leading to acantholysis. It usually begins in adulthood, with isolated cases at the extremes of life. It manifests as vesico-bullous lesions mainly in the flexural areas, which develop into erosions and crusts. Chronic lesions may form vegetative or verrucous plaques. Pruritus, a burning feeling and pain are common. It evolves with periods of remission and exacerbation, generally triggered by humidity, friction, heat, trauma and secondary infections. The diagnosis is based on clinical and histopathological criteria: marked suprabasal acantholysis, loosely joined keratinocytes, giving the appearance of a “dilapidated brick wall”, with a few dyskeratotic cells. The acantholysis affects the epidermis and spares the adnexal epithelia, which helps in the differential diagnosis with pemphigus vulgaris. Direct immunofluorescence is negative. The main differential diagnoses are Darier disease, pemphigus vegetans, intertrigo, contact dermatitis, and inverse psoriasis. There is no cure and the treatment is challenging, including measures to control heat, sweat and friction, topical medications (corticosteroids, calcineurin inhibitors, antibiotics), systemic medications (antibiotics, corticosteroids, immunosuppressants, retinoids and immunobiologicals) and procedures such as botulinum toxin, laser and surgery. There is a lack of controlled clinical trials to support the choice of the best treatment.

## Introduction

Hailey-Hailey disease (HHD), also called familial benign pemphigus, is a rare genodermatosis, with an autosomal dominant inheritance pattern, characterized by compromised adhesion between epidermal keratinocytes.^1^ As a consequence, the formation of vesicles, bullae, erosions and maceration occurs mainly in the intertriginous areas, in a chronic and recurrent form. Diagnosis is based on clinical and histopathological characteristics and the treatment is challenging.^2^

This Continuing Medical Education article presents a review of the pathogenesis, clinical picture, diagnostic methods and therapeutic options for this disease, which has great impact on patients quality of life.

## History

The history of Hailey-Hailey disease dates back to 1939, when the condition was first described by two American dermatologist brothers, Hugh Edward and William Howard Hailey. They identified a unique familial bullous disorder characterized by erythematous, exudative, crusted papules that appeared in intertriginous areas of the body.^3^

The Hailey brothers published two case studies involving family members affected by the disease. One year later, the same authors described 22 cases of two families distributed over four generations with similar characteristics.^3^ Some authors even considered the newly described condition as a variant of Darier’s disease or epidermolysis bullosa,^4^ which was elucidated after the molecular understanding of the pathophysiology. Initially, they called it “familial benign pemphigus” because of its similarity to pemphigus vulgaris. However, the term “Hailey-Hailey disease” has gained popularity and is widely used to refer to this disease.^5^

## Epidemiology

The prevalence of HHD is unknown; however, it is estimated to be similar to that of Darier’s disease, which is estimated to be around 1/50,000, with no gender or race predilection.^1^ The disease occurs at two peaks: at the end of adolescence and between the third and fourth decades of life. There are cases reported in children aged three and five years (with a documented mutation of the *ATP2C1* gene), with a suggestive histopathological pattern.^6^, ^7^, ^8^

## Etiopathogenesis

HHD is caused by a heterozygous mutation of the *ATP2C1* gene,^6^ located on the long arm of chromosome 3 (3q21-q24),^9^ which encodes the hSPA1C protein.^1^ It is an autosomal dominant genodermatosis with complete penetrance and variable expressivity.^1^ In 15%‒30% of cases, this mutation is sporadic and those with the disease have no family history.^10^ The homozygous mutation is lethal in animals.^11^

In addition to the Mendelian inheritance pattern, postzygotic mutations can affect one of the alleles of a normal embryo, causing exclusively segmental lesions – type 1 mosaicism – or promoting the loss of the normal allele of an embryo that has the germline mutation in heterozygosity, resulting in the early segmental manifestation of the disease, later associated with the classic Hailey-Hailey condition – type 2 mosaicism.^12^ Patients with type 1 segmental disease are at risk of transmitting it to their offspring when there is gonadal mosaicism. In type 2 mosaicism, the chance of transmitting the mutation is 50%.^13^

At least 250 missense, nonsense, frameshift and splice-site mutations^14^ have been described.^15^ It is believed that nonsense alterations determine the reduction or absence of hSPA1C synthesis due to mRNA degradation (haploinsufficiency).^15^ Missense mutations can cause changes in the structure, location and stability of the hSPCA1 protein, with a decrease in its expression and functionality.^15^

The transmembrane protein hSPA1C occurs in all tissues, with preferential expression in keratinocytes.^16^ It acts as an ATPase transporting Ca^2+^ and Mn^2+^ in the Golgi apparatus, promoting calcium influx into this organelle and reducing its cytoplasmic level.^11^ Mutations of the *ATP2C1* gene alter this gradient, leading to cytosolic accumulation of Ca^2+^ with subsequent: (1) modification of junctional protein synthesis leading to acantholysis; (2) reduction in mitochondrial adenosine triphosphate, with disorganization of the actin fibers that constitute the adherens junction; (3) increased oxidative stress and reactive oxygen species, affecting the proliferation and differentiation of keratinocytes.^1^

The exclusive involvement of the skin in HHD seems to be related to the predominant expression of the hSPCA1 protein in keratinocytes, whereas other tissues have other Ca^2+^ transport proteins.^16^ The disturbance of calcium homeostasis also interferes with the differentiation of keratinocytes and the expression of profilaggrin and lipids, favoring the loss of integrity of the skin barrier.^1^ Recent studies suggest that conformational mutations in the hSPCA1 protein increase its affinity for Ca^2+^ and selectively reduce Mn^2+^ transport, contributing to the disease pathogenesis.^16^

Btadini et al. also evaluated the expression of *ATP2C1* in fibroblasts from patients with Hailey-Hailey disease after heat exposure. The authors demonstrated a reduced expression of *ATP2C1* mRNA in fibroblasts obtained from diseased skin when compared to normal fibroblasts, suggesting that an inadequate response to an increase in temperature may contribute to the loss of cell homeostasis in intertriginous areas.^17^

## Clinical aspects

**Figure 1:**
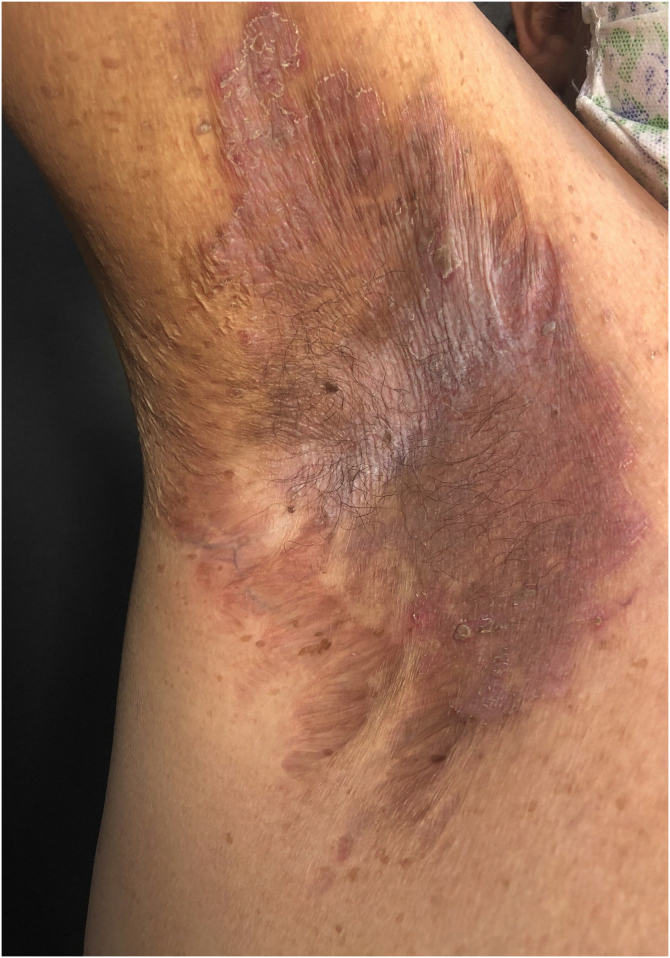
Classic involvement in Hailey-Hailey disease: vesico-pustular and eroded circinate lesion with central hyperchromia in the axillary region.

**Figure 2:**
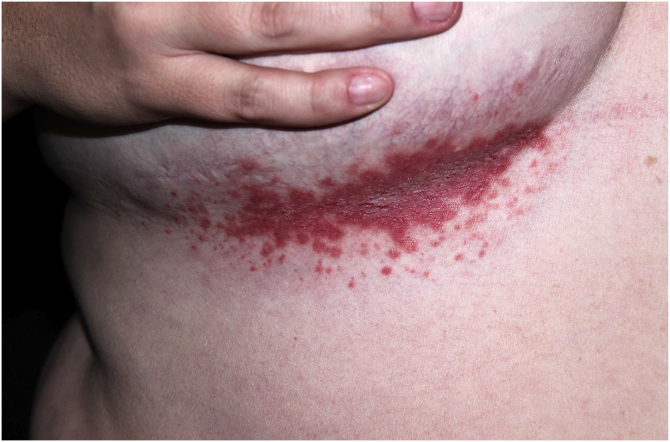
Macerated erythematous plaque with linear erosions in the inframammary region.

**Figure 3:**
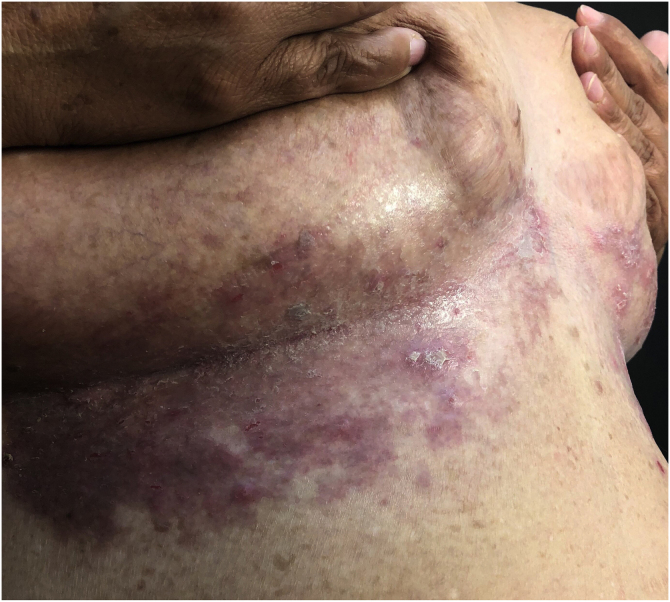
Macerated violaceous plaque with discrete lichenification in the inframammary region.

**Figure 4:**
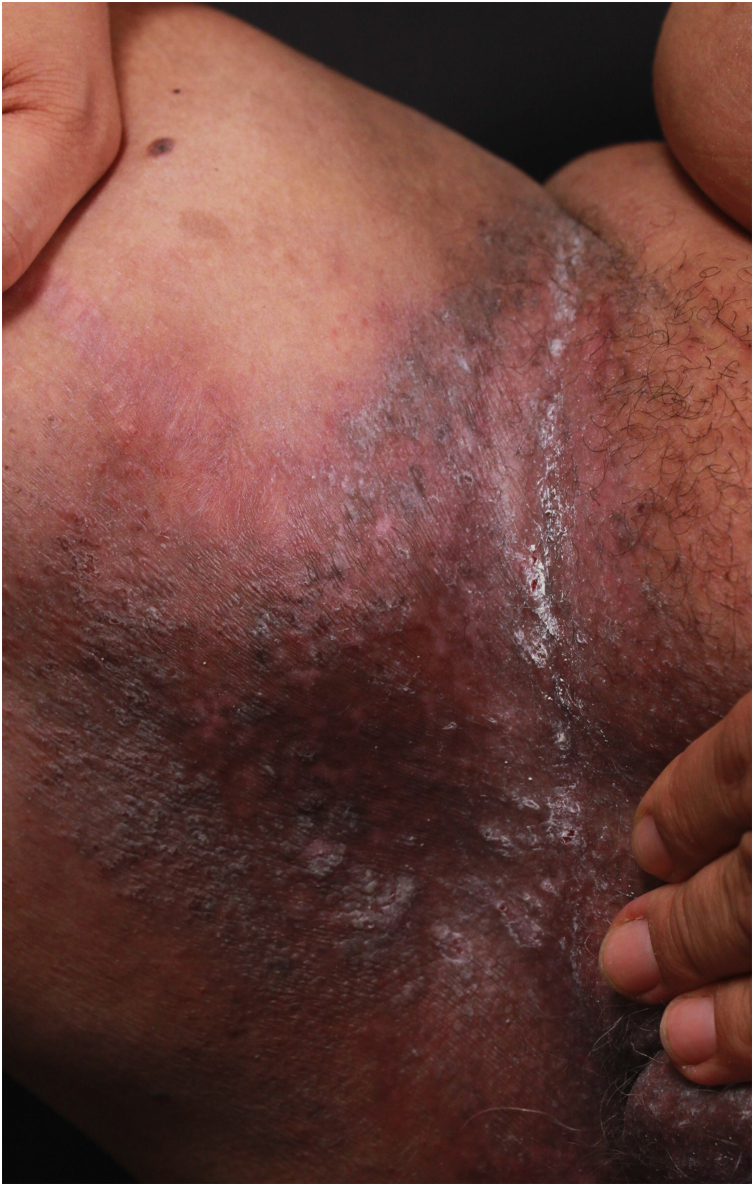
Hyperchromic plaque with lichenified edges and hyperchromic center in the inguinal region.

**Figure 5:**
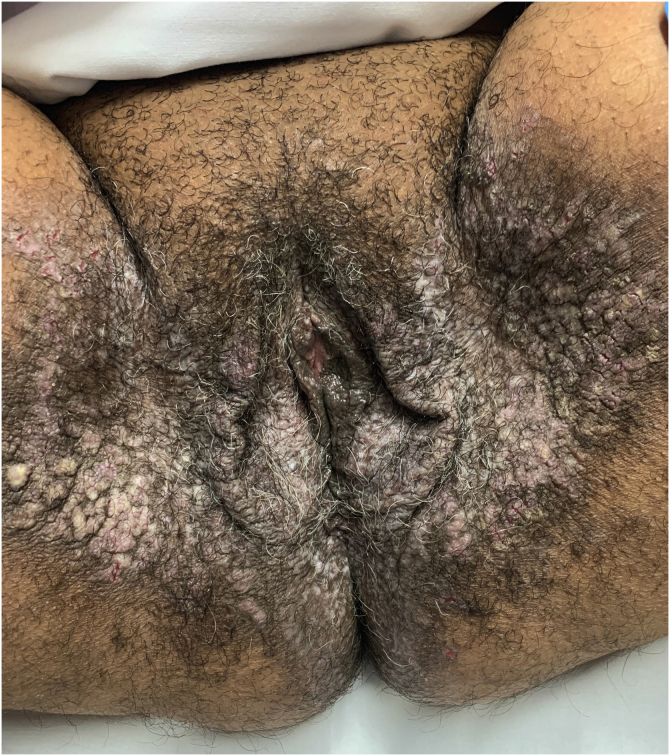
Confluent papules in a verrucous plaque on the vulvar and inguinal region.

**Figure 6:**
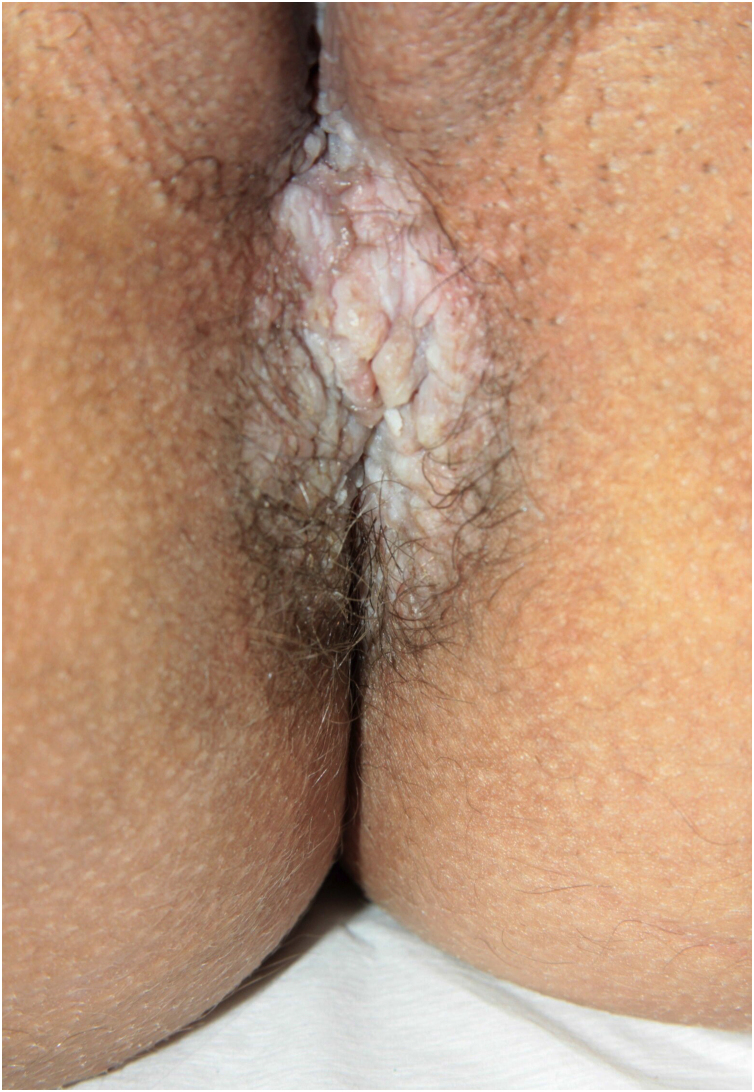
Vegetative perianal plaque.

**Figure 7:**
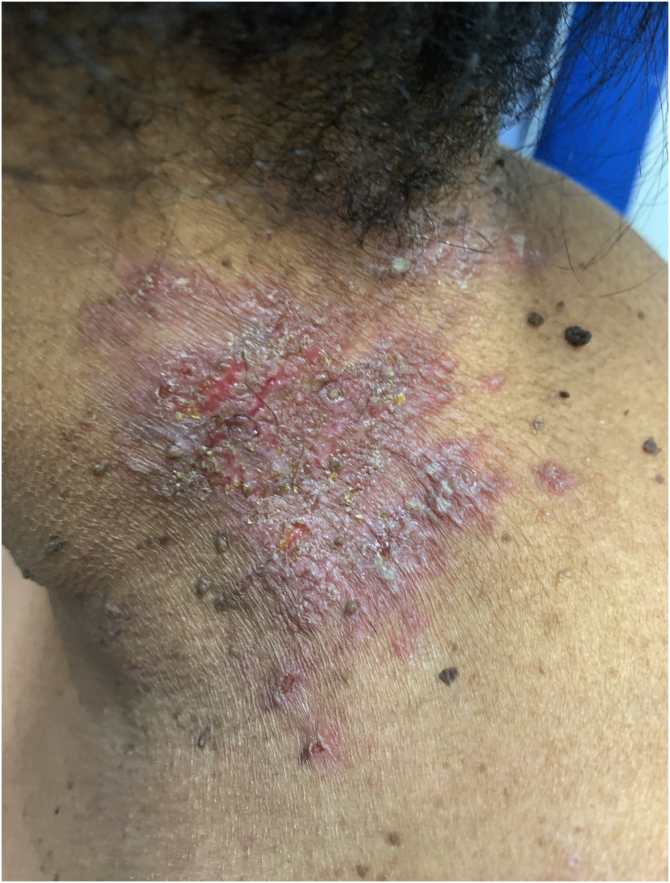
Grouped vesico-pustules with erythema, linear erosions and meliceric crusts in the cervical region.

**Figure 8:**
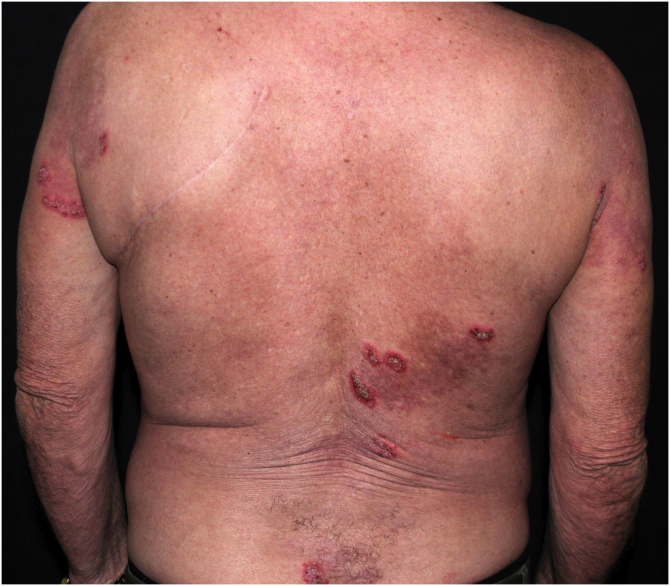
Circinate erythematous plaques with peripheral vesicles, erosions and desquamation on the back and arms.

**Figure 9:**
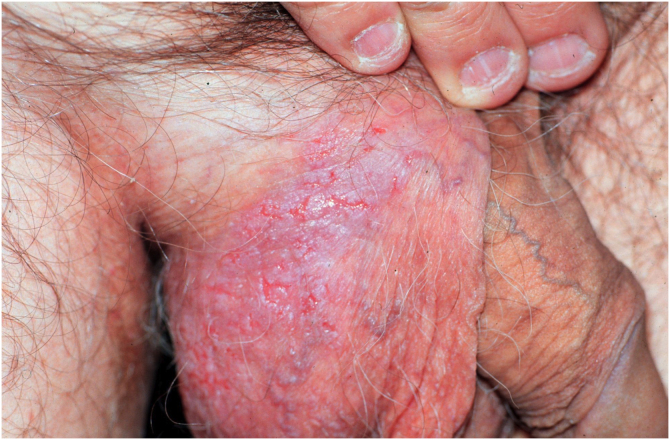
Other clinical manifestations of Hailey-Hailey disease: (A) verrucous plaque with linear erosions in the scrotal region. Also note the leukonychia striata.

HHD is a chronic dermatosis that manifests itself in adulthood (2nd to 4th decades), with isolated cases at the extremes of life. The clinical course is characterized by periods of remission and exacerbation, with reports of possible improvement with age.^10^ It manifests as vesico-bullous lesions distributed preferentially in the flexural areas, such as the axillary, inframammary in women and inguinal-crural region (Figure 1, Figure 2, Figure 3, Figure 4), in addition to the posterior cervical, genital and perianal regions (Figure 5, Figure 6, Figure 7). The lesions are symmetrical, with bilateral distribution, and may develop into erosions and crusts, or present centrifugal progression with an active circinate edge and central resolution with dyschromia (Fig. 8). Chronic lesions can form vegetative or verrucous plaques, depending on the location, which show small linear erosions characteristic of the disease (Fig. 9). Less commonly affected areas include the scalp, antecubital and popliteal fossae, in addition to the vulvar region, which may be the only manifestation.^9^ Pruritus, a burning sensation and pain are common symptoms that impair patients quality of life. Nail alterations are found in up to 70% of patients and are characterized by whitish longitudinal bands.^10^

The segmental clinical forms, previously referred to as type 1 and type 2 mosaicism (see etiopathogenesis), are respectively responsible for type 1 and type 2 segmental manifestations. Type 1 shows exclusively segmental lesions following Blaschko’s lines. In type 2, the lesions show two topographic patterns, the segmental one of early onset followed later by the classic intertriginous pattern in adulthood. When the intertriginous condition manifests itself, worsening of the segmental lesions occurs due to overlapping.^18^

The disease can undergo acute exacerbations triggered by humidity, friction, heat, trauma and secondary bacterial, fungal and herpetic viral infections,^2^ with possible progression to Kaposi varicelliform eruption due to dissemination of the type I/II herpes simplex virus.^19^ In infectious exacerbations, lesions may present exudation with a fetid odor. There are reports of the disease worsening with exposure to ultraviolet radiation,^20^, ^21^ and recurrence during pregnancy.^22^

## Histopathology

**Figure 10:**
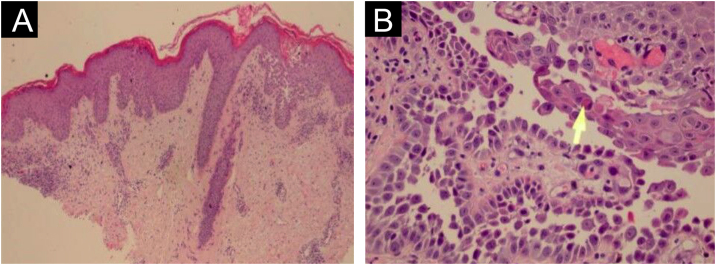
(A) Suprabasal acantholytic dyskeratosis in the epidermis that does not affect the follicular epithelium (Hematoxylin & eosin, ×20); (B) Detail of acantholysis (arrow) (Hematoxylin & eosin, ×100).

**Figure 11:**
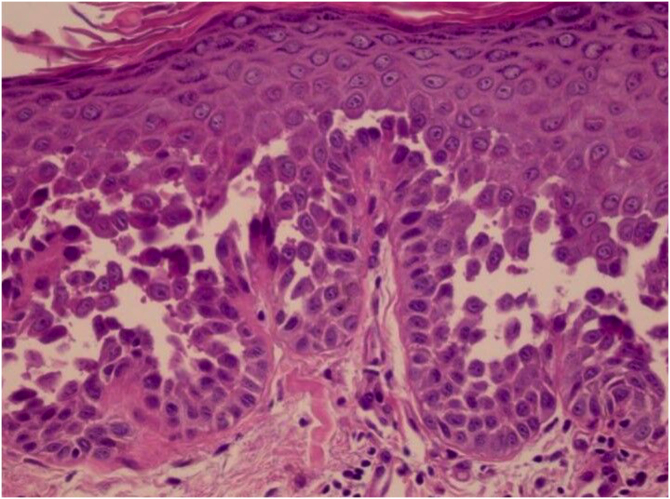
Presence of acantholytic cells filling the cavity and giving the appearance of a dilapidated brick wall (Hematoxylin & eosin, ×400).

HHD is a type of acantholytic dyskeratosis. The morphological appearance of early lesions is that of a suprabasal bulla with acantholytic cells outlining the basal layer and filling the area of detachment (Fig. 10A).^23^ The characteristic finding is marked acantholysis, interspersed with dyskeratotic cells (Fig. 10B), loosely joined, giving the appearance of a “dilapidated brick wall” (also described as an “unplastered wall” as shown in Fig. 11). This acantholysis usually affects the epidermis and spares the adnexal epithelia, which helps in the differential diagnosis with pemphigus vulgaris, where acantholysis affects all epithelia.^24^ Sometimes eosinophilic compact hyperkeratosis with parakeratosis and acanthosis can also be observed. Direct immunofluorescence is negative. Immunohistochemical studies have confirmed that desmosomal proteins and glycoproteins are synthesized in Hailey-Hailey disease. In damaged skin, cytoplasmic immunoexpression of desmogleins 2 and 3, desmoplakins I and II and desmocollins is observed.^25^

## Diagnosis

The diagnosis of HHD is based on characteristic clinical findings and histopathological confirmation.

Recent publications have attempted to establish typical dermoscopic patterns for HHD.^26^, ^27^, ^28^, ^29^ The detection of the mutation in the *ATP2C1* gene through molecular biology techniques is not routinely performed but may be useful in difficult cases.^2^

## Differential diagnosis

The clinical differential diagnoses of HHD include: Darier’s disease, pemphigus vegetans, inverse psoriasis, contact dermatitis, impetigo, acanthosis nigricans, erythrasma, intertrigo (candidal or not), tinea cruris and, in the perianal region, eczematous conditions and condyloma acuminatum.^2^, ^30^

## Treatment

Due to the multifactorial nature and lack of randomized clinical studies with a high degree of scientific evidence, the treatment of HHD is challenging. Several treatment modalities have been described in reports or retrospective case series, discussed below and summarized in Table 1.

**Table 1:** Therapeutic modalities for Hailey-Hailey disease, with respective doses, level of evidence and recommendation.

Treatment	Dose	Level of evidence	Recommendation
Topical corticoids	Low potency twice a day	II (large cross-sectional study, case series)	First line of treatment
Topical calcineurin inhibitors	Tacrolimus 0.1% ointment	III (case reports, small case studies)	Alternative to topical corticosteroids, and/or maintenance
	Pimecrolimus 1% cream 1x to 2x/day		
Topical antibiotics	Clindamycin 1% cream or gel	III (case reports, retrospective series)	Second line of treatment in combination with topical corticosteroids
	Gentamicin 0.1% cream		
	Mupirocin 2% cream, 2x to 4x/day		
Topical calcipotriol	2x/day	III (case reports, small case studies)	Alternative in case of topical corticosteroid failure
Topical 5% 5-fluorouracil	3x/week for three months	III (one case report)	More studies are needed
	1x/week for three months		
Topical iodine cadexomer	Unavailable	III (one case report)	More studies are needed
Oral antibiotics	Doxycycline 100 mg for three months	II (large case series)	Second line of treatment
	Minocycline 200 mg for two weeks, 100 mg for two months		
Dapsone	100 to 200 mg/day	III (small case series)	More studies are needed
	Maintenance 50 mg/day		
Oral retinoids	Acitretin 10 to 25 mg/day	III (case report, small case series)	Acitretin – third line of treatment
	Etretinate 25 to 60 mg/day		
	Alitretinoin 30 mg/day		
Oral corticoids	Prednisone 0.5 mg/kg	III (few patients mentioned in case series)	Use is not recommended
Cyclosporine	2.5 mg/kg/day for three weeks and weaning in six months	III (case report)	Contradictory results
			More studies are needed
Methotrexate	7.5 to 15 mg/week	III (case report)	Contradictory results
			More studies are needed
Thalidomide	300 mg/day and maintenance 50 mg for six months	III (case report)	More studies are needed
Azathioprine	Dose unavailable; for three months	III (one case report)	More studies are needed
Immunobiologicals	Etanercept- 25 mg/week for one month, 50 mg/week for Six months, 75 mg/week	III (case report, small case series)	More studies are needed
	Dupilumab – 600 mg loading dose, 300 mg in alternate weeks		
Botulinum toxin	50 to 500 UI per area, depending on the surface	II (systematic review, retrospective case studies, large case series, case reports)	First line of treatment
Laser therapy	CO2 continuous mode	II (systematic review, retrospective case study)	Second line of treatment
Photodynamic therapy	One to three sessions every three weeks	III (case report, small case series)	More studies are needed
Surgical treatments	Dermabrasion	II (large case series)	Second or third line of treatment for refractory cases
	Surgical excision		
Oral anticholinergics	Glycopyrrolate 1 mg/day	III (case report)	More studies are needed
	Oxybutynin 5 mg/day		
Naltrexone	1.5 to 6.25 mg/day	II (large and small case series)	Second line of treatment

### General non-pharmacological measures

Local conditions such as excess sweat, heat and friction can aggravate HHD lesions. Therefore, lifestyle changes that improve these factors bring benefits to all patients. Avoiding hot environments and sweating when possible, keeping the lesions clean and dry, cleaning them with antiseptic solutions, wearing appropriate clothing that reduces friction and heat, and losing weight are always recommended.^31^

### Topical treatments

#### Topical corticosteroids

They are considered the first line of treatment for HHD due to their inflammation-modulating effect. In a cross-sectional study with 58 patients, 86% showed good response after treatment with topical corticosteroids.^10^ Early application can stop the progression at the onset of lesions. Exacerbations are preferably treated with low-potency topical corticosteroids in short courses to reduce complications. Although more effective, high-potency corticosteroids should be avoided due to the risk of side effects such as atrophy, striae, telangiectasia and systemic absorption, especially in intertriginous areas. It is possible to consider the use of intralesional corticosteroids in lesions refractory to treatment with topical corticosteroids.^32^

#### Topical calcineurin inhibitors

They are indicated for long-term control of inflammation, due to the good safety profile for use in intertriginous areas when compared to topical corticosteroids, although they have less scientific evidence regarding efficacy since such results only come from isolated reports and small case series. Tacrolimus 0.1% ointment has better penetration than pimecrolimus 1% cream due to the vehicle. It can be used as monotherapy once or twice a day, with remission after two to four weeks of treatment, replacing or alternating with topical corticosteroids after initial control of the lesions, or in association with systemic treatments in refractory cases. There are reports of recurrence of lesions after discontinuation of calcineurin inhibitors.^31^, ^33^

#### Topical antibiotics

Bacterial colonization and infection by *Staphylococcus* and *Streptococcus* are factors that modify HHD by triggering the appearance of lesions or delaying the response to treatment. Therefore, topical antibiotics and antiseptics help to manage the disease. There are reports of good response to the use of clindamycin 1% cream or gel, gentamicin 0.1% cream or mupirocin 2% cream, two to four times a day for two to four weeks, associated with washing the lesions with an antiseptic solution. There is evidence that topical aminoglycosides, especially gentamicin, are capable of inducing translational reading of mutations in genetic diseases and, therefore, would benefit patients with HHD, since approximately 20% of the pathogenic mutations in this disease lead to a premature stop codon.^31^, ^34^

#### Alternative topical agents (calcipotriol, 5-fluorouracil, iodine cadexomer)

They have a low level of scientific evidence regarding their effectiveness, but are considered potential treatments as new studies emerge.

Calcitriol or calcipotriol (1,25-dihydroxyvitamin D3) is the active metabolite of vitamin D, capable of inducing keratinocyte differentiation through a calcium regulatory effect. There are reports of its efficacy when used twice a day for one month, with complete remission for three months, and a superior response to topical betamethasone in treating half of the lesion in the same patient. It is an alternative treatment after failure with the use of topical corticosteroids.^35^

There is a report of successful treatment with topical 5-fluoracil cream applied three times a week for three months, followed by weekly applications for another three months, and complete remission three months after the end of treatment, with no recurrence within one year. However, more studies are needed to prove its real effectiveness.^36^

Iodine cadexomer has antimicrobial, anti-inflammatory and skin exudate absorptive properties, necessary in cases of HHD. A case report showed complete lesion improvement after its use for ten months. However, more evidence is needed to prove this effect.^37^

### Systemic treatments

#### Oral antibiotics

They are useful in the management of HHD, especially in association with topical treatments, and are considered second-line therapy. The effectiveness of antibiotics such as erythromycin and penicillin has already been demonstrated in case reports. Tetracyclines are also effective in treating HHD. Doxycycline 100 mg daily for three months, followed by the use of 50 mg as a maintenance dose, achieved complete improvement in five of six patients in case reports. Minocycline 100 to 200 mg per day was also effective in controlling the lesions after two months, with no recurrence in a three-month follow-up. It is important to emphasize that tetracyclines have an anti-inflammatory effect in addition to the desired antimicrobial one.^38^, ^39^

#### Dapsone

This is a sulfone with anti-inflammatory and antimicrobial effects, rarely used in HHD. There are reports of improvement in lesions and pruritus in three cases after using dapsone 100 to 200 mg per day, followed by a maintenance dose of 50 mg per day. However, like many other treatments mentioned before, more evidence is needed to validate its effectiveness.^40^

#### Oral retinoids

They are considered the third-line therapy in HHD. The likely mechanisms of action are regulation of calcium homeostasis and keratinocyte differentiation in the epidermis. Several case reports have demonstrated the effectiveness of acitretin 10 to 25 mg per day for at least five months and etretinate 25 to 60 mg per day for two to six weeks. For female patients of reproductive age, alitretinoin 30 mg per day was effective as monotherapy in one case report and prevented the recurrence of lesions after discontinuation of oral prednisolone in another report. However, isotretinoin has no demonstrated efficacy in HHD.^41^, ^42^

#### Oral corticosteroids

The use of oral corticosteroids in the treatment of HHD is not recommended due to high rates of recurrence and rebound effect after drug discontinuation, except when absolutely necessary to control severe cases or in low doses as maintenance therapy. Therefore, oral corticosteroids can control the disease in the short term but should be avoided due to the risk of exacerbation after drug discontinuation.^10^

#### Other immunosuppressants (cyclosporine, methotrexate, thalidomide, azathioprine)

Several immunosuppressants have already been tested in refractory HHD with contradictory results. Therefore, they are considered exception treatments, and more studies are needed.

Cyclosporine promotes the regulation of intracellular calcium levels and pro-inflammatory cytokine levels in keratinocytes. Like corticosteroids, it offers rapid improvement in refractory cases of HHD, but with recurrence after drug discontinuation. There is a report of complete remission after low-dose cyclosporine (2.5 mg/kg/day) during three weeks, with slow weaning over six months and remission for two years, with small relapses that responded to topical tacrolimus. Nephrotoxicity and high blood pressure are possible side effects.^43^

Methotrexate at a dose of 7.5 to 15 mg per week showed complete response in case reports after three months, sustained for two years after medication withdrawal. However, there are more reports of treatment failure than success regarding the use of methotrexate in HHD.^44^

Thalidomide is considered an option in severe cases refractory to other treatments. There is a report of rapid improvement after one week with thalidomide 300 mg per day in a patient who hadn’t responded previously to dapsone and intravenous corticosteroids. Thus, thalidomide may be useful for a specific group of patients, after the in-depth discussion of long-term side effects.^45^

Azathioprine was recently reported as treatment for HHD in combination with topical antibiotics with good response within five days and partial remission within three months.^46^

#### Immunobiologicals

There are reports of controversial responses after using etanercept (anti-TNFα) with weekly doses between 25 and 50 mg. However, most reports claim against any positive effect of anti-TNFα in HHD.^47^

Recently, the use of dupilumab (anti-interleukins 4 and 13) as treatment for HHD was reported. A series of three cases demonstrated an important response after two months of treatment, with a sustained response for up to 25 months. However, another series of three cases did not show the same sustained response.^48^, ^49^

There is also a report of a patient with multiple sclerosis and HHD who was treated with ocrelizumab (humanized anti-CD20 monoclonal antibody) and showed control of skin lesions.^50^

### Medical procedures

#### Botulinum toxin

Widely used, botulinum toxin can be considered as an adjuvant treatment of choice in the management of HHD. It promotes the reduction in sweat production by blocking the release of acetylcholine in the eccrine glands nerve endings. Reduced sweating protects against bacterial colonization and subsequent exacerbation of the disease. In a recent systematic review, among 38 patients treated with botulinum toxin, only one did not respond, while the others showed partial or complete improvement. No side effects were reported. However, there is no standardization regarding the type of botulinum toxin to be used and its dilution; besides the dose applied per area is extremely variable (50 to 500 IU, depending on the surface). Some reports recommend the application of botulinum toxin at a dose of 50 IU per area as the first-line treatment for HHD.^51^, ^52^

#### Laser therapy

Due to the recurrent nature of HHD and the scarcity of proven effective treatments, laser therapy has been explored, with more reports and better responses on CO_2_ laser in continuous mode. In a systematic review that included 23 patients treated with CO_2_ laser, ten patients showed no recurrence, 12 had an improvement of less than 50% and one patient had no improvement with follow-up varying from four to 144 months. There were few adverse effects, such as depigmentation and scars in two cases. The probable mechanism of action consists of ablation of the epidermis with preservation of most of the dermis and adnexal structures, which induces re-epithelialization and resolution of the lesions.^53^

There are case reports using lasers such as erbium YAG and pulsed dye laser, with variable responses and degrees of recurrence, so more studies are needed. There is no evidence of the benefit of using diode lasers in the treatment of HHD.^54^

#### Photodynamic therapy

Although the results are conflicting and the procedure is of difficult access in clinical practice, photodynamic therapy can be considered in patients with disease refractory to multiple previous treatments, such as CO_2_ laser, surgery and retinoid use. A series of eight cases showed complete cure without recurrence in three patients and partial improvement with decreased frequency and intensity of recurrence in the other five cases.^55^

#### Surgical treatments

The surgical approach is indicated for localized HHD refractory to conventional treatments, either due to lack of efficacy or only temporary response. Although it offers permanent results, surgical procedures involve high morbidity.

Dermabrasion leads to the destruction of the epidermis and superficial dermis, sparing the skin appendages, which allows re-epithelialization of the treated area. In a series of cases, dermabrasion was performed in a total of 46 regions of ten patients with HHD. Remission was observed for up to 79 months in 38 of the treated areas. Overall, the resolution rate of HHD lesions with dermabrasion is 83%.^56^

Surgical excision followed by skin grafting can be considered the only curative treatment for recalcitrant HHD and provides definitive relief of the lesions and consequent improvement in quality of life. The largest series reported eight patients treated with surgical excision followed by grafting, with complete or almost complete remission after nine years of follow-up.^57^

### New treatments

#### Oral anticholinergics

These are treatment options due to their antiperspirant action. In one case, oral glycopyrrolate 1 mg associated with topical mometasone and oral minocycline 100 mg showed good response after one month of treatment and the improvement was maintained for six months with the use of glycopyrrolate only. Another case showed significant improvement after using oxybutynin 5 mg a day.^58^

#### Naltrexone

This is an opioid antagonist indicated for the treatment of opioid dependence or intoxication at a dose of 50 to 100 mg/day. At low doses, it has been used to treat chronic inflammatory diseases, such as fibromyalgia, Crohn’s disease and HHD. Its effectiveness is explained by the presence of opioid receptors in the skin, responsible for the nociceptive and inflammatory responses associated with stress, as well as for an adequate differentiation of keratinocytes. There are numerous case series using naltrexone from 1.5 to 6.25 mg/day in HHD, with promising results (rapid response and sustained remission) and few side effects (nausea and dizziness). It is currently recommended as second-line treatment given the evidence to date and good safety profile.^59^, ^60^

#### Other emerging treatments

Pilot studies or case series reports indicate the effectiveness of the following treatments in HHD: α-melanocyte-stimulating hormone,^61^ apremilast (phosphodiesterase-4 inhibitor),^62^ magnesium chloride,^63^ oral vitamin D,^64^ ultraviolet B therapy (in generalized forms)^65^ and electron beam radiation therapy.^66^ However, these are incipient results that require greater evidence regarding their efficacy and safety.

## Evolution and prognosis

There is no cure for this genodermatosis, which generally has a chronic evolution with periods of remission and exacerbation. The main goals of treatment are to relieve pain and pruritus, reduce the risk of secondary infection, and minimize factors that trigger exacerbations. Randomized controlled clinical trials are needed to better support the best therapeutic option for each patient.

## Financial support

None declared.

## Authors’ contributions

Adriana Maria Porro: Collection, analysis and interpretation of data; drafting and editing of the manuscript or critical review of important intellectual content; critical review of the literature; approval of the final version of the manuscript.

Camila Arai Seque: Collection, analysis and interpretation of data; intellectual participation in the propaedeutic and/or therapeutic conduct of the studied cases; approval of the final version of the manuscript.

Denise Miyamoto: Collection, analysis and interpretation of data; intellectual participation in the propaedeutic and/or therapeutic conduct of the studied cases; approval of the final version of the manuscript.

Diego Vanderlei Medeiros da Nóbrega: Collection, analysis and interpretation of data; intellectual participation in the propaedeutic and/or therapeutic conduct of the studied cases; approval of the final version of the manuscript.

Milvia Maria Simões and Silva Enokihara: Collection, analysis and interpretation of data; intellectual participation in the propaedeutic and/or therapeutic conduct of the studied cases; approval of the final version of the manuscript.

Claudia Giuli Santi: Collection, analysis and interpretation of data; intellectual participation in the propaedeutic and/or therapeutic conduct of he studied cases; approval of the final version of the manuscript.

## Conflicts of interest

None declared.
